# Evaluation of the Blinq Vision Screener in the Detection of Amblyopia and Strabismus in Children

**DOI:** 10.1167/tvst.11.4.10

**Published:** 2022-04-13

**Authors:** Arnaud Devlieger, Abdelhakim Youssfi, Monique Cordonnier

**Affiliations:** 1 Department of Ophthalmology, Erasmus Hospital, Université Libre de Bruxelles, Brussels, Belgium

**Keywords:** pediatric, strabismus, amblyopia, prevention

## Abstract

**Purpose:**

Amblyopia is a major health problem with an estimated 2% to 4% of the population affected. Screening combined with corrective measures, such as correction of refractive error and occlusion of the dominant eye, could reduce this prevalence. A new pediatric vision scanner, the blinq (Rebion, Boston, MA), studies the foveolar quality of fixation of each eye during binocular viewing. Based on the initial premise that poor quality foveolar or non-foveolar fixation is indicative of strabismus and, potentially of amblyopia, this study evaluates the effectiveness of the blinq screening device in detecting these two conditions compared to a standard ophthalmic examination (Gold Standard) based on the recommendations of the American Association for Pediatric Ophthalmology and Strabismus.

**Material and Methods:**

A prospective study was performed on a total of 101 children between 2 and 8 years of age. These children were offered a test by the blinq screening device before a standard ophthalmological examination in the ophthalmology department of the Erasmus Hospital in Brussels, Belgium. The two tests were then compared.

**Results:**

In a pediatric population heightened with amblyopia and strabismus (prevalence of 33.4%) and based on the Gold Standard Examination, the blinq device showed a specificity of 73.1% (95% confidence interval [CI] = 60.9%–83.2%) with a sensitivity of 91.2% (95% CI = 76.3%–98.1) to detect these conditions. The positive and negative predictive values were 63.3% (95% CI = 53.4%–72.2%) and 94.2% (95% CI = 84.6%–98%) respectively. The positive likelihood ratio (LR+) was 3.39 (95% CI = 2.26–5.11) for a negative likelihood ratio of 0.12 (95% CI = 0.04–0.36).

**Conclusions:**

The blinq device has good sensitivity, but insufficient specificity to be used alone in the first line of screening. Whereas other devices on the market detect risk factors that may lead to amblyopia, the blinq pediatric vision scanner detects poor foveolar fixation and strabismus, giving it a potential advantage in sensitivity to directly detect strabismus, including microstrabismus. The blinq does not detect refractive abnormalities, however, and will therefore need to be improved in the future to be used alone in pediatric vision screening.

**Translational Relevance:**

The blinq device detects visual axis alignment abnormalities with potential impact in the early detection of strabismus and subsequent associated amblyopia.

## Introduction

Amblyopia, or “lazy eye” for the public, manifests itself by a decrease in visual acuity, generally unilateral, occurring during the maturation of the cerebral structures handling visual function. This condition is a major public health problem, with between two and four percent of the European population affected.^1^^,^^2^

The main causes of amblyopia can be classified into three categories according to their origin. One category is amblyopia induced by strabismus, which is predominant in children under 3 years of age. A second category is amblyopia induced by refractive anomalies, unilateral with anisometropia, but which can also be bilateral with pronounced astigmatism, hyperopia, or myopia, more frequently noted in children over 5 years of age; between the ages of 3 and 5 years, the 2 causes may be equally common. A third category is deprivation-induced amblyopia. This may be caused by anomalies that partially or totally obstruct the entry of light into the eye, including cataracts, corneal and vitreous media opacities, or ptosis within the first 5 years of life.^3^

Amblyopia may not only have psychosocial and academic effects for the child,^4^^,^^5^ but is also one of the most common causes of vision loss in the pediatric population.^6^ Simple measures based on a combination of an effective and early screening campaign with basic corrective measures may prevent visual loss from amblyopia. These various corrective measures include patching of the dominant eye, or its penalization by pharmacological (atropine drops) or optical (overcorrection) means, or by surgery if an opacity blocks the pupil.^3^^,^^7^^,^^8^

The assessment of amblyopia and its risk factors is through direct examination of visual acuity defects via subjective sensory tests, along with a series of examinations measuring refraction and ocular alignment (see Materials and Methods). However, such testing requires not only an older child with sufficient cooperation for the examination, but also trained personnel to perform it.

To overcome such problems for the direct detection of sensory amblyopia, indirect methods have been developed over the years. These methods are based on the objective measurement of refractive abnormalities and strabismus that could lead to amblyopia in children. Methods frequently used rely on autorefractors such as the Retinomax (Righton, Tokyo, Japan) or the Suresight (Welch Allyn Medical Products, Skaneateles Falls, NY).^9^

For the detection of microstrabismus, a Gracis 6 prism diopter biprism (composed of 2 base-opposite horizontal prisms of 6 PD placed vertically over one another) test associated with a Lang or other stereoscopic tests may be used.

After passing such indirect screening tests, patients suspected of having risk factors for amblyopia are referred to an ophthalmologist for further examination.

However, these indirect techniques lead to problems of over-referral. Indeed, 30% to 70% of refractive abnormalities perceived as amblyogenic will not necessarily lead to amblyopia.^10^^,^^11^ These false positive results cause unnecessary additional workloads for ophthalmologists.

Recently, a new pediatric vision scanner called the blinq (Rebion, Boston, MA) has come on the market. Akin to autorefractors already in use, the blinq has the advantage of being noninvasive and portable, and could be used as a new screening tool by nonmedical staff, such as nurses or orthoptists. Unlike autorefractors, however, it does not measure refraction, but detects even small angles of strabismus (one degree or greater) by directly monitoring the foveolar quality of fixation. According to some studies,^12^^,^^13^ poor fixation is associated with some forms of amblyopia.

Indeed, the key concept of the blinq device rests upon the birefringent property of the Henle fibers surrounding the fovea; the detection of birefringent patterns confirms or disproves aligned foveal fixation of the images. Circular polarized light is sent through the device with a set frequency: if centered foveal fixation occurs, the Henle fibers, due to their radial arrangement around the fovea and their birefringence property, will reflect this polarized light with a specific frequency. If fixation is not centered or stable on the fovea, the reflected frequency is different and will result in a frequency difference between the two eyes.^14^

Since its commercialization in 2018, no studies have been conducted on this device in Europe. Moreover, the few independent studies performed have obtained different results.^10^^,^^15^^–^^18^ Therefore, the primary aim of this study is to evaluate the effectiveness of the device in detecting strabismus, with potential attendant amblyopia, compared to a standard ophthalmic examination performed by qualified personnel. The second aim is to determine whether blinq has a place as a first-line preschool/school vision screening tool.

## Materials and Methods

### Study Design

This is a single-center prospective interventional study conducted from February 2021 to March 2021 in the ophthalmology department of the Erasmus Hospital in Brussels, Belgium. The protocol of this study was submitted to and approved by the Ethics Committee of the Erasmus Hospital (EC P2020/589), respecting the principles of the Declaration of Helsinki.

### Participants

Children seen in the ophthalmology department were invited to participate in the study. A total of 101 children were recruited consecutively. Recruitment criteria were age between 2 and 8 years, absence of any known neurodevelopmental disorder, and no cycloplegic eye drop administration prior to screening with the device.

The purpose of the study was explained to the parents with a letter of consent provided and signed by the parents. A letter of assent explaining the study in simplified terms was also signed.

Whereas previously published studies using the blinq or earlier prototypes included between 100 and 300 patients,^10^^,^^15^^,^^16^ our sample size was arbitrarily determined. Given the general prevalence of amblyopia mentioned above, and the fact that the Erasmus Hospital is a tertiary referral center, we assumed that there would be enough children with amblyopia among the 101 examined to allow for a satisfactory statistical analysis.

### Data Collection

To evaluate the effectiveness of the blinq device, each of the participants was first screened with the device by the same independent assessor. Then, within 2 hours, the child received a full ophthalmic screening by the same pediatric ophthalmologist (author A.Y.). In doing so, the examination was blinded and the specialist was not influenced by the result provided by the screening device.

### Pediatric Vision Screening Device

The blinq allows a test to be completed in 5 to 10 seconds when performed correctly. The child is placed in a darkened room, about 40 cm from the device held by the examiner, and must stare at a “smiley face” target around which polarized light is streamed. During this time, the device takes 5 to 10 scans and calculates a binocularity score. This score has been set at 60% by the manufacturer, with a higher score indicating good fixation. The device will give either a “PASS” result in case of good binocular fixation, or a “REFER” result in case of poor fixation (<60%), and “TIME-OUT” in case of an unachievable test due to poor cooperation or poor execution of the examination. Unfeasible tests were considered in the statistical analysis.

### Gold Standard Examination

The Gold Standard (GS) examination was always performed by the same pediatric ophthalmologist (author A.Y.). The GS for pediatric ophthalmic screening consisted of the following series of examinations recommended by the American Association for Pediatric Ophthalmology and Strabismus (AAPOS) for the detection of amblyopia or strabismus: monocular visual acuity measurement, autorefraction without and with cycloplegia (cyclopentolate 1%), and Brückner test (comparative transpupillary illumination [red reflex] test). Strabismus was investigated by single cover testing to disclose tropia, and by Gracis biprism testing to disclose microtropia. Stereoscopic vision was also assessed, with either the Lang or TNO test.

In preverbal children, acuity was measured using preferential gaze methodology.

In all children, use of the blinq device and the GS examination took place within 2 hours.

### Definition of Amblyopia and Strabismus

A child was considered amblyopic by GS examination when the visual acuity scores of the two eyes differed by at least two LogMAR lines. Amblyopia was considered binocular when the visual acuity of the better eye was less than –0.3 LogMAR (corresponding to 5/10 visual acuity in the decimal scale).

A child was considered to have strabismus when deficient stereoscopic vision was associated with tropia via single cover testing, or if they had a positive Gracis biprism test result over one eye.

### Statistical Analysis

The statistical requirements for our database were initially checked. Children were classified as positive/negative for amblyopia and/or strabismus after the GS examination. Then, the synthetic indices for the evaluation of test performance were calculated for the screening device: sensitivity, specificity, positive and negative predictive values, and positive and negative likelihood ratios. For each of these indicators, a 95% confidence interval (CI) was calculated.

The statistics were produced using the Microsoft Excel program.

### Literature Review

The literature search for this study was based on the following databases: PubMed/MEDLINE, ScienceDirect, Cochrane Library using the “P.I.C.O.” method with the following keywords: “Vision screening,” “Amblyopia treatment,” “Amblyopia,” “Pediatric Vision Scanner,” “Pediatric Vision Screener,” “Amblyogenic factors,” “Vision screening,” “Morbidity,” and “Quality of life.”

## Results

### Demographic Characteristics

One hundred one children were recruited between February 20, 2021, and March 22, 2021. Demographic characteristics are summarized in Table 1. Mean age was 5 years and 3 months (± 1.9 SD). Parity of recruitment was respected, as 52 participants were boys (51.49%). Most examinations (*n* = 75) consisted of follow-up visits (74.3%).

**Table 1.** Demographic Analysis

	No., %
**Number of subjects**	101
**Average age**	5 y and 3 mo (SD ± 1.9 y)
**Gender**	
** Female**	49 (48.5%)
** Male**	52 (51.4%)
**Amblyopia + strabismus**	9 (8.9%)
**Amblyopia**	10 (9.9%)
**Strabismus**	15 (14.9%)
**Neither of the two**	67 (66.3%)
**First** **visit**	26 (25.7%)
**Follow-up**	75 (74.3%)

### Gold Standard Examination Results

Of the recruited sample, 34 children had amblyopia and/or strabismus. Refractive and strabismic amblyopia were present in almost equal proportions (52% refractive; see Table 1).

### Screening Device Results

As shown in Table 2, the device provided results for 93 out of the 101 children (92.1%). Fifty-two children received a PASS from blinq and 41 a REFER.

**Table 2.** Screening Device Results

	
**Pass**	52 (51.5%)
**Refer**	41 (40.6%)
**Time-out**	8 (7.9%)

The test could not be correctly performed in eight children (TIME OUT 3 times consecutively), these were considered as REFERs for statistical analysis, according to the manufacturer's recommendations, bringing the number of REFERs to 49.

### Statistical Analysis

Table 3 presents the results of the screening device versus GS examination in a two-by-two contingency table. The specificity (Sp) of blinq was 73.1% (95% CI = 60.9%–83.2%) with a sensitivity (Se) of 91.2% (95% CI = 76.3%–98.1%). The positive likelihood ratio (LR+) was 3.39 (95% CI = 2.26–5.11) for a negative likelihood ratio (LR-) of 0.12 (95% CI = 0.04–0.36). The positive predictive value (PPV) and negative predictive value (NPV) were 63.3% (95% CI = 53.4%–72.2%) and 94.2% (95% CI = 84.6%–98%), respectively.

**Table 3.** Gold Standard versus Screening Device

	Gold	Gold	
	Standard +	Standard −	Total
**Screening device REFER (+)**	31	18	**49**
**Screening device PASS (−)**	3	49	**52**
**Total**	**34**	**67**	**101**

Due to the tertiary hospital setting of this study, the prevalence of amblyopia and/or strabismus was 33.4%. A recalculation of the PPV and NPV with a prevalence of 3%, equivalent to that of the general population, gave a PPV of 9.50% (6.5%–13.6%) and an NPV of 99.6% (98.9%–99.9%).

### Unfeasibility

We observed that the majority of the unachievable performances came from children under 5 years of age with a strictly identical distribution in the presence or absence of amblyopia/strabismus. We also observed that 50% of those children had greater than 5 diopters of hyperopia (Table 4).

**Table 4.** Unfeasible

	
**Unfeasible**	8
**Age**	
** 2 y**	3
** 3 y**	2
** 5 y**	1
** 7 y**	1
** 8 y**	1
**Amblyopia/strabismus**	4
**No amblyopia/strabismus**	4
**Refractive anomalies**	
** Hyperopia 3D** ** ** **>** ** ** **0D**	3
** Hyperopia 5D** ** ** **>** ** ** **3D**	1
** Hyperopia >5D**	4

## Discussion

### Diagnostic Performance of the Screening Device

No screening test achieves 100% sensitivity and specificity. Generally, a decision is made to focus on either sensitivity or specificity, with these performances varying inversely. As the visual abnormalities of interest here are not life-threatening, it is important to have a high specificity, above 90%, on the one hand, to avoid false positive results leading to overloading ophthalmologists’ practices^9^ as well as to avoid excessive costs for the parents by unnecessary referrals.

Our study showed a very good sensitivity of 91.2% (95% CI = 76.3%–98.1%) but a rather unremarkable specificity of 73.1% (95% CI = 60.9%–83.2%). These performances are lower than those found by Loudon et al.^15^ (Se 96% and Sp 96%), by Jost et al.^16^^,^^18^ (Se 97% and Sp 90%) and by Bosque et al.^17^ (Se 100% and Sp 89%). They appear similar, however, to those of the study by Nishimura et al.^10^ (Se 41% and Sp 77%), although we noted better sensitivity. We explain these differences.

The three studies using the blinq pediatric vision scanner or earlier prototypes that noted better results used different methodologies. In the study by Loudon et al., the patient sample included children and adolescents 2 to 18 years of age. Hence, by including older children, compliance for performing vision screening was better.^19^ Nonperformers were also not counted as “REFER,” but were excluded from their study. These two differences reduce false positive results and may explain a gain in specificity. In the study by Jost et al.,^18^ selection bias was not taken into account in the contingency table results for their enriched population. Indeed, 62.6% of children had strabismus and/or amblyopia and sensitivity was therefore overestimated.^18^ In a previous study without an enriched population,^16^ sensitivity could not be meaningfully determined because of the “unable to perform” rates of 7%.^16^

The study by Bosque et al*.* described better sensitivity and specificity, but with the use of less strict criteria for defining amblyopia.^17^ Finally, in the study by Nishimura et al*.*,^11^ the blinq was not used to screen only for amblyopia and/or strabismus, but for refractive disorders as well, a purpose for which the blinq is not developed. As could be expected, this resulted in a high false negative rate and poor sensitivity in their study (41%). Such findings provide additional confirmation of how the defocus and strabismus detections should not be equated.

### Place of the Blinq in the First Line of Screening

A key issue is the age at which the child should be screened. In order to be effective against strabismus or amblyopia, it is important to detect the condition before it becomes irreversible. In the case of nonrefractive amblyopia, as shown by the ATS^20^ and MOTAS^21^ studies, treatment can be carried out up to the age of 7 years, but significantly better results are obtained when the correction is carried out before the age of 4 or 5 years. It is therefore recommended that all children be screened before this age.^3^^,^^22^^,^^23^ When we recalculate the synthetic indices for this age group (under 5 years) from our sample of 37 children, we find a sensitivity of 94.7% and a specificity of 61.1% with an LR+ of 2.44. Such an LR+ provides only a moderate gain in terms of pathology detection. It should be noted that the LR+ for our entire sample (i.e. 3.39, 95% CI = 2.26–5.11), is not much better.

In terms of current child screening programs, different recommendations exist. In the United States, the Vision in Preschoolers (VIP) study group investigated the diagnostic performance of several devices, including the Retinomax and Suresight autorefractors. They found a specificity of 90% and a sensitivity of 70%, the latter rising to 85% when using the same diagnostic criteria as our study.^24^ In Belgium, and more specifically in the Wallonia-Brussels Federation, screening by trained personnel is carried out by the “Office de la Naissance et de l'Enfance” (ONE). The screening includes an inspection (head-lids-eyes), a Gracis biprism test, a cover test, a Lang test, and Retinomax refractometry. This set of screening tests can detect strabismus, but also refractive abnormalities. The disadvantage is that these tests require trained staff and are more time-consuming (i.e. about 15 minutes compared to the 10 seconds of blinq) On the other hand, the specificity of the screening currently performed has 86%,^25^ significantly better than the blinq in a pediatric population 18 to 36 months of age.

These considerations led us to continue our present first-line screening method, not yet shifting to use of the blinq alone, because maintaining high specificity is important to have fewer children wrongly referred. This results not only in lower costs, but also in reduced anxiety for parents with an increase in the confidence and value in screening overall.^26^

The blinq does not actually detect amblyopia, but rather poor foveal fixation. The developers of the device have relied on some studies that concluded that poor fixation or microstrabismus is associated with amblyopia,^12^^,^^13^^,^^15^ but this is not categorical (corroborated by findings from Nishimura et al. also using the blinq device^11^). Forms of amblyopia exist without strabismus, and vice versa. Nonetheless, the principle upon which the blinq is based shows promise, as it can directly detect ocular misalignment unlike other devices which detect the risk factors that indirectly are linked to strabismus or amblyopia. Recent enhancements of the underlying technology by Guyton et al. permit the detection of deviations as small as 1.5 prism diopters.^14^

### Ways to Improve the Vision Screening Device

We believe that the blinq could be improved on several points. One of the main disadvantages currently is the cutoff of the binocularity score set at 60%.

We suspect that by imposing stricter conditions for positivity, the number of positive results (true and false) may be reduced and the number of negative results (true and false) increased, resulting in a decrease in sensitivity and an increase in specificity. Set at a higher cutoff, we believe the blinq could show reasonable sensitivity with better specificity, more appropriate for preschool/school vision screening.

On the other hand, for older children closer to the age limit for correction/reversal of amblyopia, it may be better to modulate the cutoff downward to obtain a better sensitivity,^27^ even at the expense of specificity. Such free choice of the cutoff point was possible in pre-commercialized versions of the vision screening device^15^^,^^28^; we believe this option should be made available again in future models.

Additionally, a system of sounds and/or lights emitted by the device, as was available in prior non-commercialized versions,^16^^,^^29^ with recently developed improvements,^14^ available in some other screening devices,^29^ could be re-introduced to increase the child's attention for target fixation. Most importantly, the re-inclusion of improved focus detection capabilities, as previously developed by Guyton et al., would constitute a crucial enhancement.^14^

At present, the blinq also suffers from poor ergonomics, requiring both hands of the examiner to be used, with a fairly high weight (2 kilograms), making it one of the heaviest and most difficult to handle devices on the market.^30^

### Strengths and Limitations of the Study

Ours was a prospective and consecutive study with all the classical advantages of this type of design. This approach, together with a blinded conduct of the GS examination and screening device, gave it added strength.

It did, however, have some limitations. The first was selection bias as our patient sample, with an increased prevalence of amblyopia and strabismus, was not representative of the general population.^1^^,^^2^ We compensated for this bias by recalculating the predictive values, with the PPV proving to be much poorer: PPV of 63.3% before correction for bias, and only 9.5% afterward.

Another limitation was the relatively small sample of patients, which implies rather wide, although nonetheless reliable, confidence intervals for our results.

A similar study conducted on a large captive population would avoid these limitations.

## Conclusion

Aiming to study the quality of fixation of each eye under binocular conditions, the blinq was intended to be an innovative and rapid method for the detection of non-binocular or poor-quality fixation, corresponding to strabismus and, for the makers of the device, amblyopia. The advantage of this instrument is that it directly detects poor foveal fixation rather than its risk factors, but the specificity remains unimpressive for this generation of the device. The underlying principle for the device is, however, attractive and an improved version has the potential to play an effective role in vision screening.
